# Association of Hypoxia-Inducible Factor-2 Alpha Gene Polymorphisms with the Risk of Hepatitis B Virus-Related Liver Disease in Guangxi Chinese: A Case-Control Study

**DOI:** 10.1371/journal.pone.0158241

**Published:** 2016-07-06

**Authors:** Liling Huang, Cunxu Liu, Yan Deng, Yanqiong Liu, Jiangyang Zhao, Xiuli Huang, Wenjun Tang, Yifan Sun, Xue Qin, Shan Li

**Affiliations:** 1 Department of Clinical Laboratory, First Affiliated Hospital of Guangxi Medical University, Nanning, Guangxi, China; 2 Department of Clinical Laboratory, Longtan Hospital of Guangxi Zhuang Autonomous Region, Liuzhou, Guangxi, China; 3 Department of Infectious Disease, Longtan Hospital of Guangxi Zhuang Autonomous Region, Liuzhou, Guangxi, China; 4 Department of Clinical Laboratory, Third Affiliated Hospital of Guangxi University of Chinese Medicine, Liuzhou, Guangxi, China; Drexel University College of Medicine, UNITED STATES

## Abstract

**Objective:**

Hypoxia-inducible factor-2 alpha (HIF-2a) plays a major role in the progression of disease, although the role of HIF-2α gene polymorphisms in hepatitis B virus (HBV)-related diseases remains elusive. The aim of this study is to determine whether HIF-2a rs13419896 and rs6715787 single-nucleotide polymorphisms (SNPs) are associated with susceptibility to chronic hepatitis B (CHB), liver cirrhosis (LC), or hepatocellular carcinoma (HCC).

**Method:**

A case-control study of 107 patients with CHB, 83 patients with LC, 234 patients with HCC, and 224 healthy control subjects was carried out, and the HIF-2a rs13419896 and rs6715787 SNPs were genotyped by polymerase chain reaction–restriction fragment length polymorphism (PCR-RFLP).

**Results:**

No significant differences were observed in the genotype or allele frequency of two HIF-2a SNPs between the cases and controls (all *p*>0.05). However, in subgroup analysis by gender, the HIF-2a rs13419896 GA and AA genotypes were significantly associated with a risk of CHB (odds ratio [OR] = 3.565, 95% confidence interval [CI] = 1.123–11.314, *p* = 0.031 and OR = 12.506, 95% CI = 1.329–117.716, *p* = 0.027) in females, and the A allele of rs13419896 was associated with a risk of CHB (OR = 2.624, 95% CI = 1.244–5.537, *p* = 0.011) and LC (OR = 2.351, 95% CI = 1.002–5.518, *p* = 0.050) in females. The rs6715787 CG genotype polymorphism may contribute to a reduced risk of LC in the Guangxi Zhuang Chinese population (OR = 0.152, 95% CI = 0.028–0.807, *p* = 0.027), as determined via subgroup analysis by ethnicity. Moreover, binary logistic regression analyses that were adjusted by drinking status indicated that the AA genotype of rs13419896 may contribute to an increased risk of LC in the non-alcohol-drinking population (OR = 3.124, 95% CI = 1.091–8.947, *p* = 0.034). In haplotype analysis, GG haplotype was significantly associated with a reduced risk of LC (OR = 0.601, 95% CI = 0.419–0.862, *p* = 0.005).

**Conclusions:**

The HIF-2a rs13419896 polymorphism is associated with an increased risk of CHB and LC in the Guangxi Chinese population, especially in females and in the non-alcohol-drinking population, while the HIF-2a gene rs6715787 polymorphism is associated with a decreased risk of LC in the Guangxi Zhuang population.

## Introduction

Chronic hepatitis B virus (HBV) infection is a major public health problem throughout the world, particularly in sub-Saharan Africa and in most of Asia [1]. Chronic HBV infection can lead to chronic hepatitis B (CHB) and liver cirrhosis(LC); moreover, it may eventually increase susceptibility to hepatocellular carcinoma (HCC) [2]. CHB, LC, and HCC are three progressive stages of acute and chronic HBV infection, which represent a complex biological process where the molecular and cellular mechanisms of pathogenesis remain unknown [3]. HCC is a multifactorial malignant tumor involving a complex interplay between the genetic and environmental. It has been revealed that many risk factors affecting HCC, such as HBV infection, hepatitis C virus (HCV) infection, aflatoxin exposure, smoking, alcohol drinking, and non-alcoholic fatty liver disease (NAFLD) [4, 5], which are mainly external factors. The internal factors mainly include immunity, inflammation, and host genetic susceptibility (e.g., single nucleotide polymorphisms [SNPs]). Although genetic susceptibility to HCC is relatively weak, it has also been found to be associated with many gene polymorphisms in the progression of HCC [6–11]. For instance, hypoxia-inducible factor (HIF)-2a gene polymorphism may play a major role.

Tian et al. discovered HIF-2a in endothelial cells in 1997; it is also called endothelial PASprotein-1 (EPAS1), HIF-like factor (HLF), and HIF-related factor (HRF) [12]. It is located on chromosome 2p21-p16, as a heterodimer transcription factor with a composition of “a” and “β” subunits. The “a” subunit was sensitive to oxygen, which has a unique oxygen-dependent degradation domain (ODDD) and determines the biological activities of HIF; this can be activated by a lack of oxygen, inflammatory cytokines, and tumor mutation. HIF was first identified for its role in erythropoietin regulation [13], but it was later discovered to also regulate genes involved in glycolysis, angiogenesis, cell differentiation, apoptosis, and other cellular pathways [14]. Three kinds of HIF family subtypes have been identified: namely HIF-l, HIF-2, and HIF-3. Among these, HIF-1 and HIF-2 have been considered to be the most important regulators of cellular responses to hypoxia [15]. Hypoxia and HIF-a can promote dedifferentiation in a variety of cells, including cancer cells [16, 17]. Studies have confirmed the relationship between hypoxia and tumor invasiveness [18], which has complex mechanics that may involve cytotoxic resistance, a decline in deoxyribonucleic acid (DNA) repair ability, insensitivity to radiation, the promotion of blood vessel formation, and the increase of metastasis potential [4, 5, 19]. It was speculated for a long time that HIFs are associated with tumor angiogenesis, and this relationship became clear after the discovery of hypoxia promotion via the expression of vascular endothelial growth factor (VEGF) [20, 21]. Hohnquist-Mengelbier et al. [22] suggested that HIF-1 adjusts the expression of VEGF in the acute stage of oxygen restriction, while it is mainly regulated by HIF-2 in the process of long-term anoxia. HIF-1a and HIF-2a amino acids are 48% homologous, and both identify and combine the same DNA, but they regulate different downstream genes. HIF-la has been widely studied in clinical cancer cases. Initially, it was considered to be associated with tumor invasion, while HIF-2a overexpression has been demonstrated to be associated with tumor metastasis and poor prognosis for the past few years [23, 24]. It is probable that HIF-2a can serve as an effective biomarker for the early diagnosis and prognosis of tumors. Furthermore, Hepatitis B viral X protein (HBx)—a key protein of the HBV genome—has been identified as enhancing the stability of the HIF-2a protein through binding to the von Hippel-Lindau protein (pVHL) and activating the NF- κ B signaling pathway. This leads to an overexpression of HIF-2 target genes [25], which may play an important role in hepatocarcinogenesis. However, few studies have investigated the correlations between HIF-2a gene polymorphisms and HBV-related disease.

Genetic polymorphisms are considered the main genetic elements involved in the development of various kinds of diseases [26]. To date, several studies have reported associations between HIF-2a gene polymorphisms and renal cell carcinoma [27], prostate cancer [28], lungadenocarcinoma [29], non-small-cell lung cancer [30], acute mountainsickness [31], and maximum metabolic performance in elite endurance athletes [32]. However, HIF-2a gene polymorphisms’ involvement in HBV-related diseases remains elusive. Moreover, the latest study by Yanqiong Liu et al. [33] estimated the association between HIF-1a rs11549465 and rs11549467 gene polymorphisms and the risk of HBV-related HCC. They found the CG haplotype to be associated with a significantly increased susceptibility to HCC, whereas the CA haplotype was found to be associated with a significantly decreased risk of HCC in a Chinese population. We hypothesized that the HIF-2a gene polymorphisms also influence one's susceptibility to HBV-related disease in a Chinese population. In the present study, we selected two HIF-2a gene tag SNPs (rs13419896 and rs6715787), referring to previous research, which interrogated the NT-022184.14 sequence of the NCBI database with Hapview software, and we conducted a case-control study to explore the associations between the two SNP polymorphisms and HBV-related disease in a Chinese population.

## Materials and Methods

### Study subjects

A total of 648 subjects were recruited in this case-control study, including 107 patients with CHB, 83 patients with HBV-related LC, 234 patients with HBV-related HCC, and 224 healthy controls. All subjects were recruited at the First Affiliated Hospital of Guangxi Medical University from 1 June to 1 October 2014. The patients in the case group had tested positive for the hepatitis B surface antigen (HBsAg). Patients were included in the CHB group and LC group following the prevention guide of CHB diagnostic criteria (2010, China), while they were included in the HCC group following the standard of hepatocellular carcinoma diagnosis and treatment criteria (2011, China). To control for the effects of potential confounders, healthy participants were individually matched to the cases based on gender and age (±5 years).

The patients infected with other hepatitis viruses, such as hepatitis C or hepatitis E, as well as those with liver diseases caused by non-viral infection, were excluded. None of the subjects had received treatment (antivirals, surgery, radiation, or chemotherapy) before seeing a doctor.

The study protocol was approved by the Ethics Committee of the First Affiliated Hospital, Guangxi Medical University (S1 File), and written informed consent was obtained from all participants after a full explanation of the study.

### DNA extraction and SNP genotyping

**Fig 1:**
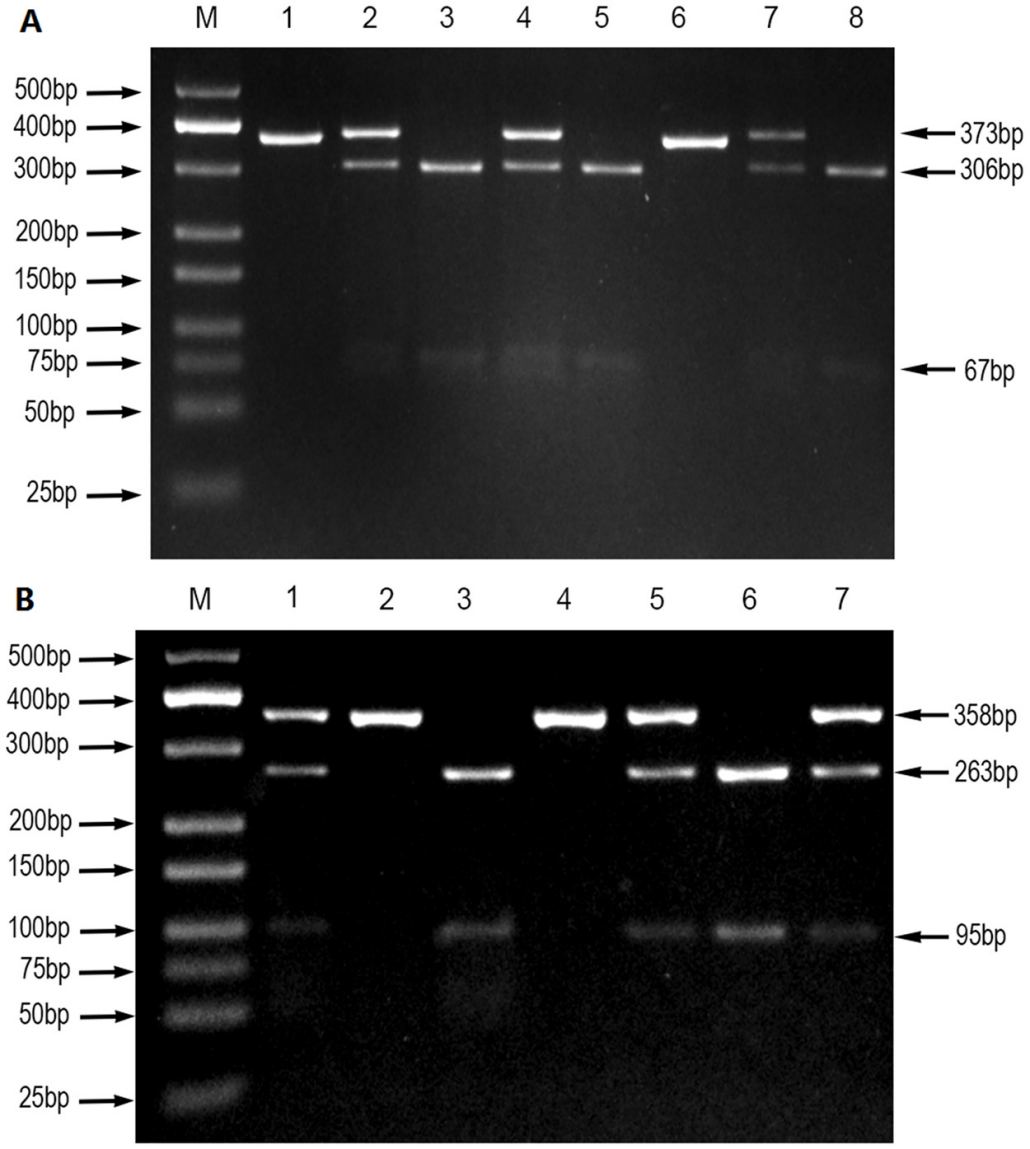
PCR-RFLP assay for analyzing the rs13419896 and rs6715787 polymorphisms of the HIF-2a gene. (A) rs13419896- lane M shows DNA marker; lanes 1 and 6 show AA genotype; lanes 2, 4, and 7 show GA genotype; lanes 3, 5, and 8 show GG genotype. (B) rs6715787- lane M shows DNA marker; lanes 2 and 4 show GG genotype; lanes 3 and 6 show CC genotype; lanes 1, 5, and 7 show CG genotype.

All operations were carried out in strict accordance with the standard operating procedure. Genomic DNA was extracted from venous blood using the standard phenol-chloroform method. The genotyping of HIF-2a rs13419896 and rs6715787 SNPs was performed via polymerase chain reaction–restriction fragment length polymorphism (PCR-RFLP). Polymerase chain reaction (PCR) was used to amplify the target DNA, with the 5'- TTCCCTGTTCCCTCCTCCTTT-3' forward primer, 5'-TCCTACCCTGTGGTTGCCTCG-3' reverse primer of rs13419896 and 5'-TCAGTTGAGCATCCCTAACCC-3' forward primer, 5'-CCAGCCCTTGTCAGCATCTTT-3' reverse primer of rs6715787. Primer sequences and reaction conditions for the genotyping of HIF-2a polymorphisms are shown in Table 1. The PCR amplification was carried out in a final volume of 25μL of a reaction mixture, consisting of 2 μL of genomic DNA (60 ng/μL), 1 μL of forward primer (10 μmol/L), 1 μL of reverse primer (10 μmol/L), 12.5 μL of DreamTaq Green PCR Master Mix (Thermo Fisher Scientific), and 8.5 μL of nuclease-free deionized water using a Perkin-Elmer thermocycler (2700, Applied Biosystems, Foster City, CA, USA). PCR amplification products of HIF-2a genes rs13419896 and rs6715787 were digested with HaeII restriction enzymes and ECORI restriction enzymes, respectively. Their enzyme-digested products were separated by 3% agarose gel electrophoresis and visualized with an ultraviolet (UV) transilluminator. Enzyme-digested products of rs13419896 included three digested fragments of 373bp, 306bp, and 67bp (Fig 1A), and those of rs6715787 included three digested fragments of 358bp, 263bp, and 95bp (Fig 1B). To confirm the accuracy of genotyping, about 10% of specimens were selected at random for direct DNA sequencing by the Sangon Biotech Company (Shanghai, China). The results of DNA sequencing and PCR–RFLP were 100% concordant.

**Table 1:** Primer sequence and the reaction condition for genotyping HIF-2a polymorphisms.

SNPs	Primer sequence (5'-3')	Annealing temperature	Restriction enzyme	Product size (bp)
HIF-2a	Forward:TTCCCTGTTCCCTCCTCCTTT	60°C 5min	HaeII	GG:306+67
rs13419896	Reverse:TCCTACCCTGTGGTTGCCTCG		37°C for 15min	GA:373+306+67
				AA:373
HIF-2a	Forward:TCAGTTGAGCATCCCTAACCC	60°C 5min	ECORI	CC:263+95
rs6715787	Reverse:CCAGCCCTTGTCAGCATCTTT		37°C for 3h	CG:358+263+95
				GG:358

### Statistical analysis

The distribution of the general demographic and clinical features between cases and controls was evaluated using the one-way analysis of variance test and the χ2 test for continuous and categorical variables, respectively. Hardy–Weinberg equilibrium (HWE) was employed with a goodness-of-fit χ^2^ test to verify the crowd representativeness of the study subjects. The χ^2^ test and Fisher exact test were used to analyze the distribution of the genotype and allele frequencies among the cases and controls. The independent risk and protective genotypes and alleles were selected by binary logistic regression analyses adjusting for age, gender, smoking status, drinking status, ethnicity, and body mass index (BMI). Odds ratios (ORs) and 95% confidence intervals (CIs) were calculated to assess the relative risk. SHEsis online software [34] was applied for the haplotype analysis of HIF-2a gene polymorphism (http://analysis.bio-x.cn/myAnalysis.php).

All of the statistical analyses were performed using the Statistical Package for the Social Sciences (SPSS, version13.0). Statistical significance was assumed at two-sided values of *p*<0.05.

## Results

### Demographic characteristics of the study population

A total of 648 participants (224 controls and 424 cases) were recruited in this case-control study. The demographic characteristics of the study are shown in Table 2 and S2 File. We carried out comparative analysis of the four groups using the following data: age, gender, nationality, BMI, smoking, and drinking. The data in Table 2 show that the male and female sex ratios in the CHB, LC, and HCC groups were 3.1:1, 4.2:1, and 11.3:1, respectively. These results are consistent with those of a previous report [35], which indicated that the sex ratio in liver cancer roughly fluctuated between 1.4 and3.3, and the higher the incidence of liver cancer, the greater the sex ratio. Our data verify that the Guangxi region of China is an endemic area for HCC. The difference in HCC sex ratios are considered to relate to the hormones and living habits of males and females. Another previous report suggested that the onset age shows a sharp increase after age 20 and peaks at 50 years old in areas characterized by a high incidence of liver cancer in southeast Asia and on the west coast of Africa [36]. The average ages in the control, CHB, LC, and HCC groups were 46.71±7.05, 37.56±11.58, 49.30±12.07, and 48.45±11.03 years, respectively, in our study, which is also consistent with previous reports.

**Table 2:** Demographic characteristics of the study population.

Variables	Controls	CHB	*p*	LC	*p*	HCC	*p*
**Total number**	224	107		83		234	
**Gender**							
Male	125	81	<0.001	67	<0.001	215	<0.001
Female	99	26		16		19	
**Ethnicity**							
Han	95	60	0.071	47	0.087	147	<0.001
Zhuang	116	43		33		83	
Others	13	4		3		4	
**Tobacco smoking**							
Yes	72	44	0.112	42	0.003	80	0.692
No	152	63		41		154	
**Alcohol drinking**							
Yes	63	54	<0.001	31	0.127	79	0.225
No	161	53		52		155	
**Age(mean±SD)**	46.71±7.05	37.56±11.58	<0.001	49.30±12.07	0.068	48.45±11.03	0.044
**BMI(mean±SD)**	22.44±3.49	22.08±3.58	0.382	22.83±5.04	0.513	22.01±3.60	0.194

CHB, chronic hepatitis B; LC, liver cirrhosis; HCC, hepatocellular carcinoma; SD, standard deviation; BMI = body mass index.

### Association analysis of HIF-2a gene polymorphisms

The genotype and allele frequencies of the HIF-2a gene rs13419896 and rs6715787 SNPs in the control and patient groups are described in Table 3. The data on genotype frequencies among the control, CHB, LC, and HCC groups were all in agreement with the HWE. No significant differences were observed in the genotype distribution or in the allele frequency of the two SNPs between cases and controls (all *p*>0.05). To explore the impact of risk factors on CHB, LC, and HCC subjects, we conducted stratified analysis of confounding factors, such as gender, age, ethnicity, smoking, and alcohol drinking. Binary logistic regression analyses adjusted by gender are shown in Table 4, indicating that the GA and AA genotypes of rs13419896 loci in females were significantly associated with a risk of CHB (OR = 3.565, 95% CI = 1.123–11.314,*p* = 0.031 and OR = 12.506, 95% CI = 1.329–117.716, *p* = 0.027). Moreover, the A allele was also associated with a risk of CHB (OR = 2.624, 95% CI = 1.244–5.537, *p* = 0.011) and LC (OR = 2.351, 95% CI = 1.002–5.518, *p* = 0.050) in females. These results revealed that the GA and AA genotypes of rs13419896 may be risk factors for CHB in females in Guangxi, while the A allele genotype of rs13419896 may be a risk factor for CHB and LC in women in Guangxi. For the rs6715787 polymorphism, the CG genotype may contribute to a reduced risk of LC in the Guangxi Zhuang population (OR = 0.152, 95% CI = 0.028–0.807, *p* = 0.027), as shown in Table 5, suggesting that it may be a protective factor in this population. Binary logistic regression analyses adjusted by alcohol drinking status indicated that the AA genotype of rs13419896 may contribute to an increased risk of LC in the non-alcohol-drinking population (OR = 3.124, 95% CI = 1.091–8.947, *p* = 0.034) (Table 6). No significant differences were found after adjusting for age and smoking found by binary logistic regression analyses.

**Table 3:** Association analysis of HIF-2a gene polymorphisms between HBV-related patients and healthy controls.

					CHB vs. Control		LC vs. Control		HCC vs. Control	
SNPs	Control(n = 224)	CHB(n = 107)	LC(n = 83)	HCC(n = 234)	OR(95% CI)*	*P*	OR(95%CI)*	*P*	OR (95%CI)*	*P*
**rs13419896**										
**GG**	115	47	35	122	1^ref^		1 ^ref^		1 ^ref^	
**GA**	93	50	39	99	1.502(0.845–2.668)	0.165	1.333(0.760–2.339)	0.316	0.904(0.591–1.382)	0.64
**AA**	16	10	9	13	2.091(0.768–5.693)	0.149	1.916(0.741–4.954)	0.18	0.574(0.249–1.323)	0.192
**G**	323	144	109	343	1 ^ref^		1 ^ref^		1 ^ref^	
**A**	125	70	57	125	1.439(0.951–2.178)	0.085	1.344(0.899–2.010)	0.15	0.835(0.606–1.151)	0.271
**rs6715787**										
**CC**	11	4	9	6	1 ^ref^		1 ^ref^		1 ^ref^	
**CG**	84	39	32	89	1.604(0.230–11.185)	0.633	0.377(0.106–1.341)	0.132	2.461(0.608–9.972)	0.207
**GG**	129	64	42	139	2.770(0.403–19.035)	0.3	0.414(0.119–1.438)	0.165	2.952(0.740–11.78)	0.125
**C**	106	47	50	101	1 ^ref^		1 ^ref^		1 ^ref^	
**G**	342	167	116	367	1.451(0.817–2.577)	0.204	0.829(0.511–1.345)	0.448	1.261(0.866–1.835)	0.227

CHB, chronic hepatitis B; LC, liver cirrhosis; HCC, hepatocellular carcinoma; SNPs, single nucleotide polymorphisms.

*Adjusted for gender, age, ethnicity, smoking, alcohol drinking status, and BMI (body mass index) by logistic regression model.

**Table 4:** Association analysis of HIF-2a gene polymorphisms between HBV-related patients and healthy controls in female population.

	CHB vs. Control		LC vs. Control		HCC vs. Control	
SNPs	OR (95% CI)*	*P*	OR (95% CI)*	*P*	OR (95% CI)*	*P*
**rs13419896**						
**GG**	1 ^ref^		1 ^ref^		1 ^ref^	
**GA**	3.565(1.123–11.314)	0.031	2.284(0.617–8.455)	0.216	1.747(0.572–5.338)	0.328
**AA**	12.506(1.329–117.716)	0.027	4.109(0.493–34.266)	0.192	0(0)	0.999
**G**	1 ^ref^		1 ^ref^		1 ^ref^	
**A**	2.624(1.244–5.537)	0.011	2.351(1.002–5.518)	0.050	1.102(0.459–2.647)	0.828
**rs6715787**						
**CC**	1 ^ref^		1 ^ref^		1 ^ref^	
**CG**	1.338(0.054–33.406)	0.859	0.725(0.083–6.296)	0.771	0.245(0.018–3.359)	0.292
**GG**	1.183(0.048–28.934)	0.918	0.589(0.070–4.981)	0.627	0.141(0.142–1.912)	0.141
**C**	1 ^ref^		1 ^ref^		1 ^ref^	
**G**	0.893(0.385–2.069)	0.791	0.789(0.323–1.925)	0.602	0.475(0.204–1.106)	0.084

CHB, chronic hepatitis B; LC, liver cirrhosis; HCC, hepatocellular carcinoma; SNPs, single nucleotide polymorphisms.

*Adjusted for age, ethnicity, smoking and drinking status by logistic regression model.

**Table 5:** Association analysis of HIF-2a gene polymorphisms between HBV-related patients and healthy controls in Zhuang nationality population.

	CHB vs. Control		LC vs. Control		HCC vs. Control	
SNPs	OR (95% CI)*	*P*	OR (95% CI)*	*P*	OR (95% CI)*	*P*
**rs13419896**						
**GG**	1 ^ref^		1 ^ref^		1 ^ref^	
**GA**	1.793(0.689–4.663)	0.231	1.107(0.466–2.628)	0.818	0.529(0.270–1.034)	0.063
**AA**	0.524(0.070–3.918)	0.529	1.329(0.266–6.640)	0.729	0.443(0.121–1.627)	0.220
**G**	1 ^ref^		1 ^ref^		1 ^ref^	
**A**	1.121(0.568–2.213)	0.741	1.116(0.596–2.090)	0.731	0.614(0.372–1.014)	0.056
**rs6715787**						
**CC**	1 ^ref^		1 ^ref^		1 ^ref^	
**CG**	1.083(0.138–8.510)	0.939	0.152(0.028–0.807)	0.027	1.350(0.130–13.974)	0.801
**GG**	1.247(0.163–9.541)	0.831	0.211(0.044–1.018)	0.053	1.468(0.146–14.733)	0.744
**C**	1 ^ref^		1 ^ref^		1 ^ref^	
**G**	1.127(0.542–2.344)	0.749	0.728(0.376–1.409)	0.346	1.094(0.649–1.846)	0.736

CHB, chronic hepatitis B; LC, liver cirrhosis; HCC, hepatocellular carcinoma; SNPs, single nucleotide polymorphisms.

*Adjusted for age, smoking and drinking status by logistic regression model.

**Table 6:** Association analysis of HIF-2a gene polymorphisms between HBV-related patients and healthy controls in non-alcohol population.

	CHB vs. Control		LC vs. Control		HCC vs. Control	
SNPs	OR (95% CI)*	*P*	OR (95% CI)*	*P*	OR (95% CI)*	*P*
**rs13419896**						
**GG**	1 ^ref^		1 ^ref^		1 ^ref^	
**GA**	1.417(0.677–2.965)	0.355	1.317(0.647–2.678)	0.448	0.895(0.540–1.483)	0.667
**AA**	2.992(0.813–11.005)	0.099	3.124(1.091–8.947)	0.034	0.859(0.324–2.273)	0.759
**G**	1 ^ref^		1 ^ref^		1 ^ref^	
**A**	1.528(0.900–2.594)	0.117	1.596(0.989–2.577)	0.056	0.916(0.627–1.337)	0.650
**rs6715787**						
**CC**	1 ^ref^		1 ^ref^		1 ^ref^	
**CG**	4.037(0.319–51.136)	0.281	0.492(0.140–1.732)	0.269	1.089(0.282–4.199)	0.902
**GG**	4.306(0.348–53.282)	0.255	0.382(0.109–1.340)	0.133	1.117(0.295–4.225)	0.871
**C**	1 ^ref^		1 ^ref^		1 ^ref^	
**G**	1.237(0.677–2.260)	0.488	0.689(0.411–1.152)	0.155	1.035(0.688–1.556)	0.868

CHB, chronic hepatitis B; LC, liver cirrhosis; HCC hepatocellular carcinoma; SNPs, single nucleotide polymorphisms.

*Adjusted for age, smoking by logistic regression model; ref refers to the reference genotype.

### Haplotype analysis of the HIF-2α gene polymorphisms

As the haplotype-based analysis is considered to have more power than SNP genotyping does, SHEsis online software was applied for the haplotype analysis of HIF-2α gene polymorphism (rs13419896 and rs6715787). We calculated the linkage disequilibrium in both the case and the control for the two SNP sites, and the value of the lowest frequency threshold for haplotype analysis is 0.03. According to the results, a total of four haplotypes (CG, CA, GG, and GA) were derived from the observed genotypes. The haplotype distribution in the CHB, LC, and HCC patients and healthy controls are shown in Table 7. The GG haplotype was the most common haplotype, followed by GA, CG, and CA, in turn. The GG haplotype was a protective factor and significantly decreased the LC risk in the Guangxi population (OR = 0.601, 95% CI = 0.419–0.862, *p* = 0.005).

**Table 7:** Analysis of HIF-2a gene rs13419896 and rs6715787 haplotype frequencies with the risk of CHB, LC, and HCC.

					CHB vs. Control		LC vs. Control		HCC vs. Control	
Haplotype	Control	CHB	LC	HCC	OR(95% CI)*	*P*	OR(95% CI)*	*P*	OR(95%CI)*	*P*
CG	0.188	0.142	0.249	0.155	0.718(0.457–1.127)	0.149	1.440(0.942–2.200)	0.091	0.795(0.563–1.122)	0.192
CA	0.049	0.078	0.052	0.061	1.627(0.842–3.146)	0.145	1.059(0.471–2.377)	0.89	1.254(0.707–2.221)	0.438
GG	0.533	0.531	0.407	0.578	0.989(0.714–1.371)	0.948	0.601(0.419–0.862)	0.005	1.197(0.922–1.554)	0.176
GA	0.311	0.25	0.292	0.206	1.114(0.762–1.629)	0.577	1.378(0.923–2.057)	0.116	0.871(0.636–1.192)	0.387

CHB, chronic hepatitis B; LC, liver cirrhosis; HCC, hepatocellular carcinoma.

*Adjusted for sex, age, ethnicity, smoking and drinking status by logistic regression model.

### Comparison of genotype distributions with the HapMap project data

Considering that the HIF-2α rs13419896 and rs6715787 genotype frequencies might be distinct among different populations, the genotype and allele frequencies of the two polymorphisms in our control group were further compared with those populations from the Haplotype Map (HapMap) project. The data in Table 8 show that the genotype and allele frequencies of rs13419896 in our control group were similar to those in the CHB (Han Chinese in Beijing, China), JPT (Japanese in Tokyo, Japan), and YRI (Yoruba in Ibadan, Nigeria)populations, but they were significantly different from those in CEU (Utah residents with northern and western European ancestry) populations. In the current study, for the rs6715787 site, the genotype and allele distributions were similar to those in the CHB and JPT populations, but they were significantly different from those of the CEU and YRI populations. This revealed that the genotype frequencies of HIF-2α rs13419896 and rs6715787 show regional and ethnic differences.

**Table 8:** Comparison of genotype and allele frequencies in the control subjects of our study and those from the HapMap Project.

SNPs	Sample	Genotype frequency (%)				Allele Frequency (%)		
**rs13419896**		AA	GA	GG	*P*	A	G	*P*
Present study†	224	16(7.1)	93(41.5)	115(51.3)	1	125(27.9)	323(72.1)	1
CHB*	82	6(7.3)	38(46.3)	38(46.3)	0.729	50(30.5)	114(69.5)	0.545
JPT*	172	30(17.4)	68(39.5)	74(43.0)	0.006	128(37.2)	216(62.8)	0.006
CEU*	226	0(0)	4(1.8)	222(98.2)	<0.001	4(0.9)	448(99.1)	<0.001
YRI*	226	14(6.2)	104(46.0)	108(47.8)	0.612	132(29.2)	320(70.8)	0.712
**rs6715787**		CC	CG	GG	*P*	C	G	*P*
Present study†	224	11(4.9)	84(37.5)	129(57.6)	1	101(21.6)	367(78.4)	1
CHB*	82	12(14.6)	28(34.1)	42(51.2)	0.025	52(31.7)	112(68.3)	0.011
JPT*	172	12(7.0)	62(36.0)	98(57.0)	0.685	86(25.0)	258(0.75)	0.273
CEU*	226	120(53.1)	84(37.2)	22(9.7)	<0.001	324(71.7)	128(28.3)	<0.001
YRI*	226	58(25.7)	126(55.8)	42(18.6)	<0.001	242(53.5)	210(46.5)	<0.001

SNPs, single nucleotide polymorphisms; CHB, Han Chinese in Beijing, China; JPT, Japanese in Tokyo, Japan; CEU, Utah residents with northern and western European ancestry; YRI, Yoruba in Ibadan, Nigeria.

*Data from HapMap Project;

^†^Data from previous reports

## Discussion

HIF-2, an important transcription factor in the hypoxic condition, was recently recognized as controlling the activation state and key microbicidal functions of immune cells [37]. A growing body of evidence has shown that HIF-2a overexpression in primary and metastatic tumors [38], and the expression level has a positive correlation with the formation of tumor blood vessels and patient mortality[39]. A high expression of HIF-2a is closely related to some tumors and indicates a poor prognosis for patients [22, 40–43]. A previous study [25] identified that HBV enhances HIF‑2a expression through HBx binding to the pVHL and activating the NF‑κ B signaling pathway, which may play an important role in hepatocarcinogenesis. Although HIFs can undergo degradation or stabilization, polymorphisms or mutations in HIFs can also influence their activity [44]. We therefore performed this study.

This is the first case-control study to reveal whether HIF-2a rs13419896 and rs6715787 polymorphisms are associated with susceptibility to CHB, LC, or HCC in the Guangxi population. In the present study, we attempted to explore the association between the HIF-2a gene rs13419896 and rs6715787 polymorphisms and HBV-related liver disease risk in a Chinese population. As a result, we found no significant differences in the genotype distribution or allele frequency of two HIF-2a SNPs between cases and controls. However, some confounding factors, such as gender, age, smoking, drinking, ethnicity, and BMI, probably play a role in the development of HBV-related liver disease, so we stratified our population to analyze the effect of these factors. In subgroup analysis, we showed that the GA and AA genotypes and the A allele of HIF-2a rs13419896 polymorphisms were associated with an elevated risk of HBV-related liver disease in female and non-alcohol-drinking populations, respectively. For the rs6715787 polymorphism, the CG genotype may contribute to a reduced risk of LC in the Guangxi Zhuang population. No significant differences were found after adjusting for age and smoking by binary logistic regression analyses. Furthermore, haplotype analysis indicated that the GG haplotype might be a protective factor for LC in the Guangxi population. According to our latest studies [33, 45] on the association between HIF-1a genetic polymorphisms (rs11549465 and rs115494657) and the risk of HBV-related liver disease, we did not find an association between HIF-1a rs11549465 and rs11549467 polymorphisms and HBV-related liver disease. However, the HIF-1a CG and CA haplotypes might be a risk factor and a protective marker, respectively, for HBV-related HCC in the Guangxi population. As can be seen from the above, our studies suggest that HIF-1α and HIF-2α gene polymorphisms may not be important contributors in HBV-related disease, but haplotypes of these polymorphisms were observed to be significantly associated with HBV-related disease. Although HIF-1a and HIF-2a subunits are very similar in their DNA binding and dimerization domains but differ in their transactivation domains, a similar result was observed. Compared with our previous studies, the present study involves performing a more detailed subgroup analysis and has some new findings.

With regard to rs13419896, which is located within intron1 of the gene, one study by Putra et al. [30], focusing on 76 Japanese non-small-cell lung cancer patients, explored the relationships between HIF-2αgene rs13419896 polymorphism and the risk of non-small-cell lung cancer with bioinformatics analysis in 2015. The researchers showed that the A allele at rs13419896 SNP may contribute to the overexpression of HIF-2α through the alteration of the binding affinity of the activator protein-1(AP-1), especially in the presence of overexpressed c-Fos or c-Jun. Moreover, the A allele of HIF-2α SNP is associated with a poorer prognosis in lung cancer patients. A similar result was observed in the present study, where the GA and AA genotypes and the A allele of HIF-2a rs13419896 polymorphisms were associated with an elevated risk of HBV-related liver disease in subgroup analysis, which indirectly confirmed the above argument. With a larger sample size (107 CHB patients, 83 LC patients, 234HCC patients, and 224 healthy controls), our results had a much greater statistical power than did the previous study. However, our result seems unexpected because the male sex and alcohol drinking are closely related to the incidence of liver cancer [35, 46]; this is an interesting phenomenon worthy of further discussion. A previous study [29] suggested that HIF-2a rs4953354 SNP is a potentially susceptible marker for the development of lung adenocarcinoma, especially in female never-smokers. Lung cancer that has developed among never-smokers represents a unique subset of cancers. Thus, we speculated that the female and non-alcohol-drinking populations represent a unique subset of HBV-related liver disease, with a different etiology and natural history. Our data were supported, but the mechanisms underlying the high risk were not fully determined. As mentioned above, a potential explanation was that the GA and AA genotypes and the A allele of HIF-2a rs13419896 might be involved in altering the binding activity of transcription factors through NF-kB, mitogen-activated protein kinase (MAPK) and extracellular signal-regulated kinase (ERK) pathways [47–49], or through HBx binding to the pVHL and activating the NF‑κ B signaling pathway HIF signals [25], leading to an overexpression of HIF‑2a. This could increase the risk of HBV-related liver disease in female or non-alcohol-drinking populations. Another possible reason for this effect may be the limited sample size in our research. Notwithstanding, our study confirmed that HIF-2a gene rs13419896 polymorphism is associated with an elevated risk of HBV-related liver disease; studies with larger samples would further confirm these results.

With regard to rs6715787, unfortunately, no research has investigated the association of HIF-2a rs6715787 polymorphism with various diseases, except for a report on living high-exercise high-training low (HiHiLo). However, one study showed that exon2 of the HIF-2a gene is the key to the DNA binding heterodimer [50]. Since rs6715787 polymorphism is located within intron 2 of the gene, near the exon2 loci, this may affect the DNA binding heterodimer probably through the NF‑κB signaling pathway, thereby altering the expression of the HIF‑2a target gene, such as Oct4 [25]. Since relevant research data are less, the potential mechanism is still not fully clear. In our study, the CG genotype of HIF-2a rs6715787 may contribute to a reduced risk of LC in the Guangxi Zhuang population, indicating that the SNP may contribute to reducing the expression of HIF-2a and may be considered a protective marker. In contrast, a comparison of genotype distributions with the HapMap project data suggested that the CG genotype was significantly higher in the YRI population, while no significant difference in genotypes was found between the remaining populations and the current study. The CG genotype distribution exhibits regional and ethnic differences, so further studies of other ethnic populations are necessary.

A haplotype is a set of closely linked genetic markers present on one chromosome; such markers tend to be inherited together more frequently than expected by chance and could better explain the observed associations compared with each polymorphism independently. Therefore, further haplotype analysis should be performed to explore the associations between the HIF-2a gene and the risk of HBV-related liver disease. Our result indicated that the GG haplotype might be a protective factor for LC in the Guangxi population; it may induce splice-site variants [51] or affect microRNA regulation [52], which could alter the expression level of HIF-2a target genes or influence the gene-gene interaction, thus leading to a reduced risk of LC.

HIFs contribute to inflammatory functions in various other components of innate immunity, such as mast cells, dendritic cells, and epithelial cells [53], which plays a major role in the progression of disease. Indeed, HIF and its interaction with innate immunity in liver diseases have been observed [54, 55]. Toll-like receptors (TLR) play a critical role in innate immunity response, and our recent study showed that the TT genotype of TLR3 rs3775290 was associated with a decreased risk for CHB, LC, and HCC [56]. The polymorphisms of cytokines (e.g., *IL-4*, *IL-6*, *IL-17*, *IL-23*, *INF-γet al*.), which have crucial roles in innate immunity, are also related to the risk of CHB, LC, and HCC in our previous studies [3, 57–60]. On the basis of this study and our previous work, genetic variants are clearly a key factor influencing innate immunity in HBV-related diseases, suggesting that they may be useful therapeutic targets for treating immune system disorders in the development of CHB, LC, and HCC.

Our study has several limitations. First, the positive results were shown only in subgroup analysis. The potential skewing of results due to the relatively small sample size should been considered when interpreting the results. Second, the current study selected only two rather than all SNPs of the HIF-2α gene. In addition, we did not measure the serum HIF-2α levels both in patients and controls; thus, the relationship between the expression of HIF-2α and HIF-2α gene polymorphisms was unclear. Considering HIF-2a SNP as a useful clinical prognostic marker of HBV-related liver disease patients, much more extensive studies on mechanisms at the cellular and molecular levels are needed in the future. Finally, the study sample was limited to the Guangxi population. Because the genotype distribution exhibits regional and ethnic differences, further studies involving other ethnic populations are necessary for systematical study.

In conclusion, HIF-2a rs13419896 polymorphism is associated with an increased risk of CHB and LC in the Guangxi Chinese population, especially in females or non-alcohol drinkers; meanwhile, the HIF-2a gene rs6715787 polymorphism is associated with a decreased risk of LC in the Guangxi Zhuang population. In addition, the GG haplotype of the two SNPs might be a protective factor for LC in this population. An effective risk prediction model should be constructed to further clarify the connection between HIF-2a polymorphism and HBV-related liver disease.

## Supporting Information

S1 File Ethical Review Committee Approval Notice. (PDF)

S2 File Original data. (XLS)

S3 File STROBE Statement—checklist of items that should be included in reports of observational studies. (PDF)
