# Supplementary material for: Association of Hypoxia-Inducible Factor-2 Alpha Gene Polymorphisms with the Risk of Hepatitis B Virus-Related Liver Disease in Guangxi Chinese: A Case-Control Study
Source: PLoS One. 2016 Jul 6;11(7):e0158241. doi: 10.1371/journal.pone.0158241 (PMC4934873; doi:10.1371/journal.pone.0158241)
Supplement: S1 File — (PDF) [file pone.0158241.s001.pdf]

中国广西南宁市双拥路6号
No.6 Shuangyong Rd.Nanning,Guangxi,P.R.China
http://www.gxmu.edu.cn/gxmufyl/index.asp
E-mail:gxyifuyi@21cn.com
Tel:0086-771-5350895
Fax:0086-771-5350031

广西医科大学第一附属医院
THE FIRST AFFILIATED HOSPITAL OF GUANGXIMEDICAL UNIVERSITY

FIRST AFFILIATED HOSPITAL of GUANGXI MEDICAL UNIVERSITY
ETHICAL REVIEW COMMITTEE
Approval Notice

Approval Number: 2016(KY-E-016)

Title: Association of Hypoxia-inducible factor-2 alpha Gene Polymorphisms With the Risk of Hepatitis B Virus–related Liver Disease in Guangxi Chinese: A Case-Control Study

Research Contents: The aim of this study is to determine whether HIF-2a rs13419896 and rs6715787 single-nucleotide polymorphisms (SNPs) are associated with the susceptibility to chronic hepatitis B (CHB), liver cirrhosis (LC), or hepatocellular carcinoma (HCC).

Applicant: Shan Li

Application Department: Department of Clinical Laboratory, First Affiliated Hospital of Guangxi Medical University, Nanning, Guangxi, China.

Acknowledgement: This study was supported by grants from Natural Science Foundation of Guangxi （NO. 2012GXNSFAA053088）

Date of Application: 13th April, 2016

Date of Approval: 15th April, 2016

Conclusion: This project fully considered and protected the rights and interests of the study objects. It meets the criteria of Ethical Review Committee. The Medical Ethics Committee of First Affiliated Hospital of Guangxi Medical University has approved the protocol.

Signature: [signature]

Lang Li

(Vice) Director of Ethical Review Committee

First Affiliated Hospital of Guangxi Medical University

Date: April 15, 2016
